# Prospective Cohort Study Investigating the Safety and Efficacy of Ambulatory Treatment With Oral Cefuroxime-Axetil in Febrile Children With Urinary Tract Infection

**DOI:** 10.3389/fped.2018.00237

**Published:** 2018-08-31

**Authors:** Elise Hennaut, Hong P. Duong, Benedetta Chiodini, Brigitte Adams, Ksenija Lolin, Sophie Blumental, Karl M. Wissing, Khalid Ismaili

**Affiliations:** ^1^Department of Pediatric Nephrology, Hôpital Universitaire des Enfants – Reine Fabiola, Université Libre de Bruxelles, Brussels, Belgium; ^2^Department of Infectious Diseases, Hôpital Universitaire des Enfants – Reine Fabiola, Université Libre de Bruxelles, Brussels, Belgium; ^3^Department of Nephrology, Universitair Ziekenhuis Brussel, Vrije Universiteit Brussel, Brussels, Belgium

**Keywords:** urinary tract infection, oral treatment, children, uropathogens, *Escherichia coli*, antibiotic resistance, urine culture

## Abstract

**Aims:** To assess the safety and efficacy of ambulatory oral cefuroxime-axetil treatment in children presenting with first febrile urinary tract infection (UTI) in terms of resolution of fever, antibiotics tolerance, bacterial resistance, and loss to ambulatory follow-up.

**Methods:** Two-year prospective single-center evaluation of the local protocol of oral ambulatory treatment of children presenting first febrile urinary tract infection (UTI).

**Results:** From October 2013 to October 2015, 82 children were treated ambulatory with oral cefuroxime-axetil. The median age was 8 months. When analyzing those 82 children treated orally, 51 (62%) completed oral treatment, 14 (17%) missed their scheduled follow-up visits (3 patients at day 2 and 11 patients at week 2), and 17 (21%) were switched to IV therapy for the following reasons: vomiting in 9, persistent fever in 5, antibiotic resistance in 2 and bacteremia in 1. Six children (8%) presented recurrent UTI after a median of 5 months of follow-up.

**Conclusions:** This 2-year evaluation suggests that oral treatment with cefuroxime-axetil in febrile UTI is feasible but should be implemented with caution. Home-treated children require reevaluation during treatment since 21% of our cohort had to be temporarily switched to parenteral therapy and 17% did not attend scheduled follow-up visits during oral treatment.

## Introduction

Urinary tract infection (UTI) is one of the most common infections diagnosed in pediatric patients (1). Early treatment with appropriate antibiotics is essential to reduce the risk of renal scarring which can lead to the development of long term renal damage (2). In almost all cases of UTI, empirical antimicrobial treatment is initiated before the laboratory results of urine culture become available. Nonetheless, there is no consensus on the most effective empirical antibiotic regimen or route of administration (3). In the past, most authorities recommended initiating antibiotic therapy by parenteral route (4). However, in the last decade American and European guidelines started to suggest initial treatment with oral antibiotics for children older than two or three months of age (5–7). Oral treatment has potential advantages, such as the relative ease of administration which does not require hospital admission. Yet, these guidelines might be inappropriate in certain settings. First, febrile children unable to retain oral fluids and medications need to be hospitalized and treated by parenteral route. The same is true for families at risk of noncompliance. Second, the sensitivity patterns to antibiotics may show significant geographical variation (8). Therefore, appropriateness of treatment guidelines has to be evaluated at the local level. Finally, in four prospective and randomized studies which documented the efficacy of oral antibiotics in UTI children, oral treatment therapy was either administrated at the emergency department (9–11) or during hospitalization (12), so that their results need to be confirmed in cohorts of children treated as outpatients.

In spite of these limitations, we started to implement the guidelines on oral treatment for children with febrile UTI from October 2013. The aim of this study was to evaluate the efficacy and safety of the ambulatory oral treatment with cefuroxime-axetil during a two-year period in terms of resolution of fever, ability to retain oral antibiotics, the adequacy of initial empirical antibiotic therapy based on antibiotic sensitivity testing, and loss to follow-up.

## Patients and methods

### Patients

UTI was suspected and urine tests requested in febrile children (≥38°C), with at least two of the following criteria: leukocytosis ≥15,000/mm^3^; CRP ≥ 40 mg/L; known uropathy; signs of systemic infection; vomiting and poor feeding in infants; and back pain and chills in older children (13).

For every child screened for UTI, a fresh urine sample was sent to the local laboratory for dipstick and automated flow cytometer analyses as well as quantitative urine culture. For children younger than 24 months, urine samples were obtained by supra-pubic aspiration or bladder catheterization. For older children, samples were obtained by clean catch (mid-stream urine) or bladder catheterization.

### Definition

#### Diagnosis of UTI required a positive urinalysis and urine culture

A positive urinalysis was defined as a trace or greater result for leukocyte esterase and/or nitrite on dipstick (Medi-test Combi 11; Macherey-Nagel, Düren, Germany), and/or the presence of ≥35 leukocytes/μL of uncentrifuged urine using the automated flow cytometer Sysmex UF-100 (Merck-Eurolab, Leuven, Belgium) (14). The laboratory staff was blinded to clinical information. In case of a positive urinalysis urine culture was done and antibiotic treatment started.

UTI was confirmed in those children having growth of ≥50,000 colony-forming units/ml of a single pathogen. In urine samples obtained by supra-pubic aspiration, any growth of enteric Gram-negative pathogens was considered significant (5). Characterization of the pathogens and antibiotic susceptibility testing was performed according to standardized procedures in the laboratory of the Department of Microbiology of our institution.

### Parenteral vs. oral treatment

**Figure 1:**
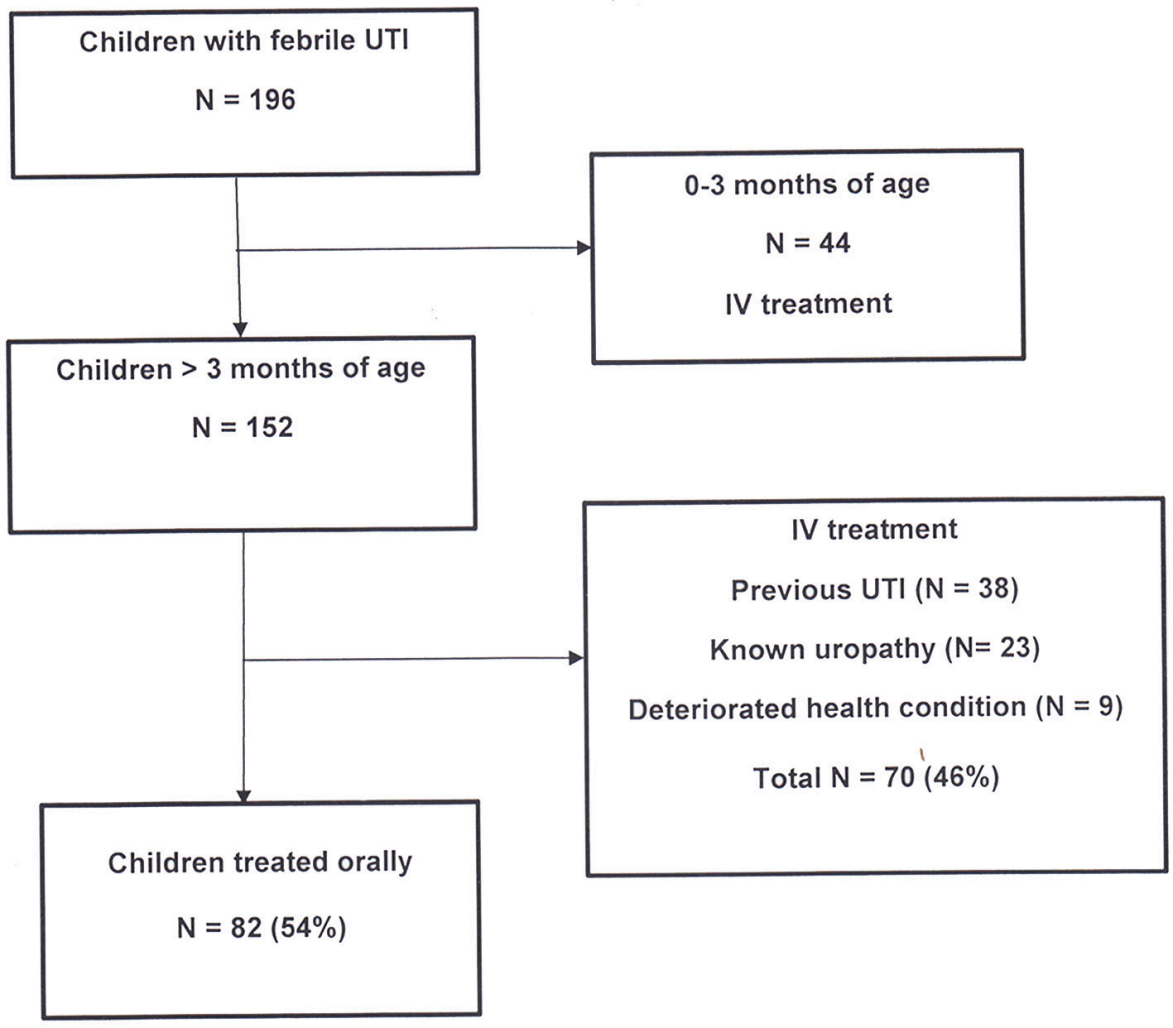
Flow diagram of inclusion criteria for oral treatment in febrile children with UTI.

All febrile patients with suspected UTI were treated according to our new local antibiotic protocol (Figure 1). A parenteral therapy was administered in children younger than 3 months, those with signs of systemic infection, vomiting or poor feeding, and in children with a history of previous febrile UTI or known uropathy. The antibiotic therapy by the parenteral route was as follows: cefotaxime and amikacin in young infants (0–3 months of age), amikacin alone when aged > 3 months, and ampicillin and amikacin in children with previous febrile UTI or known uropathy. Treatment was adapted to results of antibiotic sensitivity testing and switched to oral route after 48 h of apyrexia. The total duration of treatment was of 14 days.

Oral treatment was prescribed to children >3 months, in good health condition, without clinical or biological signs of sepsis, and with no history of febrile UTI or known uropathy. Cefuroxime-axetil 50 mg/kg/day was given in three divided doses for 14 days. According to our protocol, children treated orally were not hospitalized, but had to be reevaluated in our outpatient clinic 48 h later, in order to verify the resolution of fever (<38°C), antibiotic oral intake tolerance, and the adequacy of treatment based on antibiotic sensitivity testing results. If the outpatient clinic was unavailable (weekends and holidays), the children were seen in the emergency department. A final visit at the nephrology consultation was planned at the end of oral treatment in order to assess health condition, fever, tolerance and compliance during the whole period of antibiotic intake.

### Ethics

The study protocol was reviewed and approved by the Ethics Committee of our institution (Document number: CEH47/13). The study was designed as a quality control of treatments used in routine clinical practice in patients admitted with a suspicion of UTI in our institution. Written informed consent was therefore not required from the parents of children who contributed data to the present study but parents were informed that the data of children might be used for a clinical study in case UTI was confirmed. To guarantee data confidentiality, data were anonymized, birthdates deleted after the age had been calculated and the data file conserved on a password protected laptop computer.

### Statistics

Categorical data are shown as proportions and expressed as percentage. Continuous data are shown as medians with interquartile range (IQR). For categorical data, comparisons were made using the Fisher's exact test or Pearson's chi-square test, as appropriate. Mann-Whitney U test was used for continuous variables without normal distribution.

## Results

### Patient characteristics

During this two-year study period, 196 consecutive febrile children (208 episodes) were treated for proven UTI (positive cultures) in our hospital in Brussels. All children <3 months of age (*N* = 44) were treated by parenteral antibiotics. Among the remaining 152 children, 82 patients (54%) were treated as outpatients with oral cefuroxime-axetil and 70 (46%) were hospitalized for parenteral treatment (Figure 1).

The clinical characteristics and outcomes of the 82 patients treated orally are shown in Table 1. The median age of these patients was 8 months (IQR: 3–33). Sixty-five patients (79%) were girls. The median temperature at entry was 39.0°C (IQR: 38.6–39.7) and the median level of CRP was 50 mg/L (IQR: 17–92).

**Table 1:** Characteristics of children with febrile UTI treated with oral antibiotics.

	**Total *N* = 82**
**Gender**	
Girls	65 (79%)
Boys	17 (21%)
Age (months) (Median, IQR)	8 (3–33)
Temperature°C (Median, IQR)	39.0 (38.6–39.7)
CRP mg/L (Median, IQR)	50 (17–92)
**Uropathogens**	
*E. coli*	73 (89%)
*Proteus mirabilis*	7 (9%)
*Enterococcus faecalis*	1 (1%)
*Klebsiella pneumoniae*	1 (1%)
*ESBL*	1 (1%)
Follow up (months) (median, IQR)	14 (6–23)
Recurrence of febrile UTI	6 (7%)

### Uropathogens and antibiotic resistance

In children treated orally, the most common pathogens isolated were gram-negative bacteria, which grew in 81 (99%) of all positive cultures. The frequencies of the pathogens were as follows: *E. coli* in 73 children (89%), *Proteus mirabilis* in 7 (9%), and *Enterococcus faecalis* and *Klebsiella pneumoniae* in 1 child each (1%) (Table 1). All these pathogens were sensitive to cefuroxime-axetil, except for two: a gram-positive strain (*Enterococcus faecalis)*, and an extended-spectrum beta-lactamases (*ESBL*) producing *Enterobacteriaceae*.

### Clinical evolution (Figure 2)

At the first visit after two days of treatment, three patients (4%) were lost to follow-up. The remaining 79 children (96%) have been reevaluated in the outpatient clinic. Sixty-two (75%) of children continued oral treatment, while 17 patients (21%) were subsequently hospitalized and switched to intravenous treatment for the following reasons: vomiting in 9, persistent fever in 5, antibiotic resistance in 2 and bacteremia in 1. The last three children also had persistent fever.

**Figure 2:**
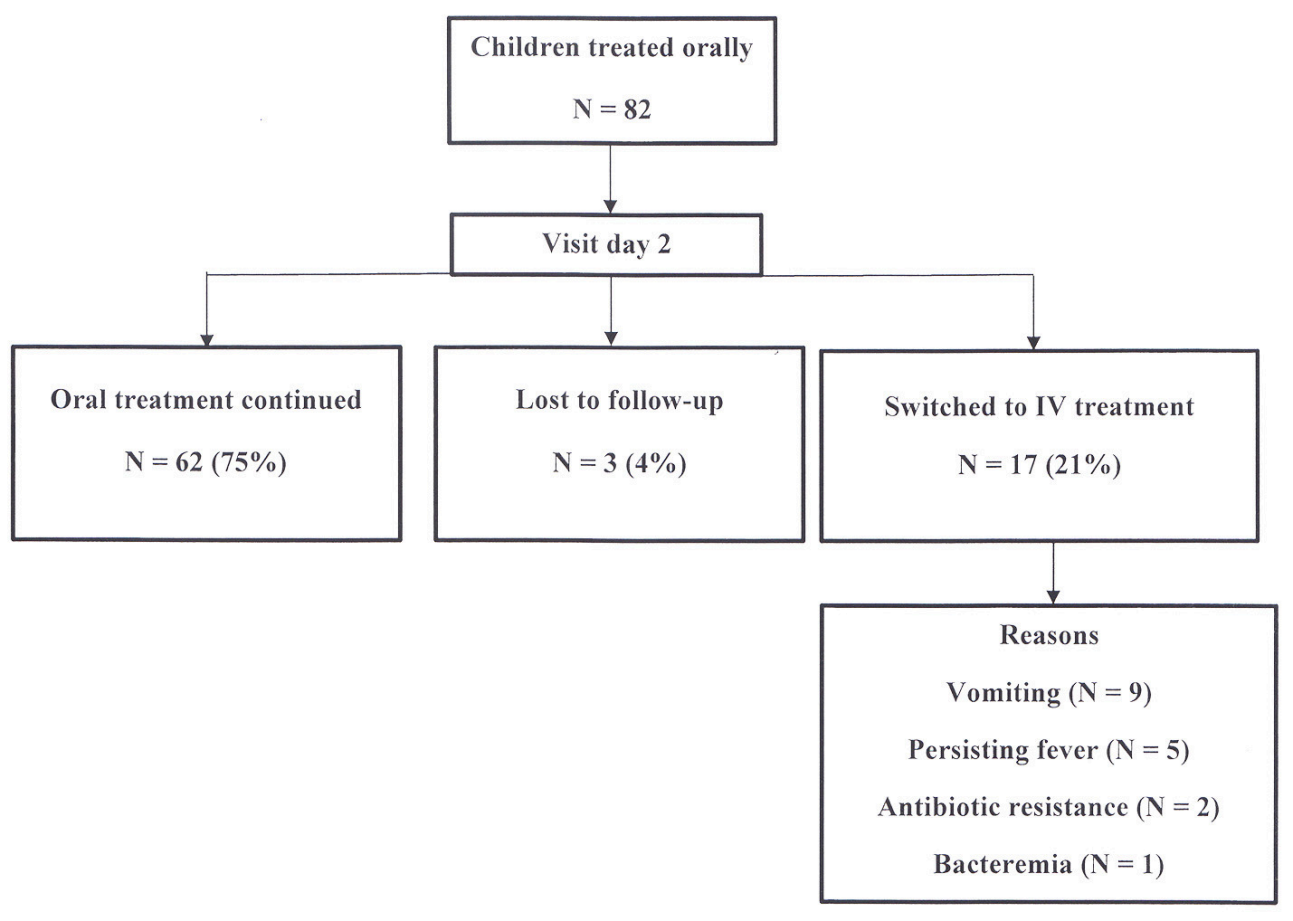
Evolution of children treated orally seen on day 2.

**Figure 3:**
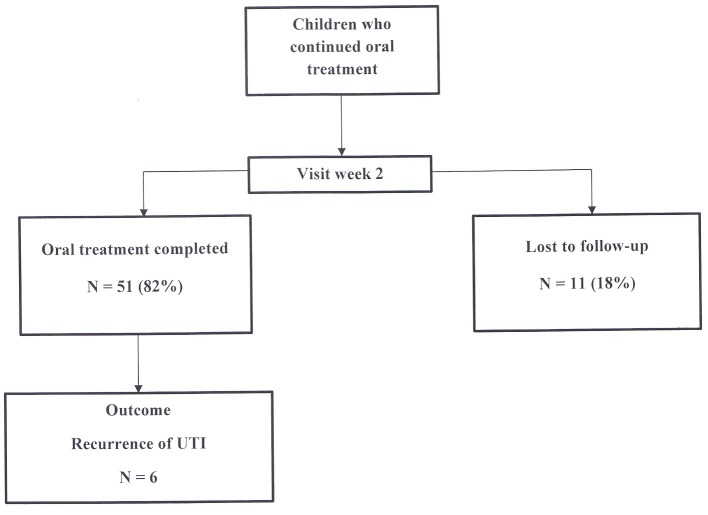
Evolution of children treated orally seen on week 2.

A final visit at the nephrology consultation was planned 2 weeks after the start of oral treatment (Figure 3). Among the 62 children who continued oral treatment, 11 (18%) children were lost to follow-up, while 51 (82%) were assessed for health condition, antibiotic tolerance and compliance to treatment. All patients were afebrile. Antibiotic intake was correctly completed in all of them.

When analyzing the whole cohort of the 82 children treated initially with oral antibiotics, 51 (62%) completed oral treatment, 17 (21%) children were switched to IV therapy and 14 (17%) children were lost to follow-up during the two-week treatment duration (3 patients at day 2 and 11 patients at week 2).

### Follow-up

The median duration of the follow-up was 14 months (IQR: 6–23). Six children had recurrence of UTI during follow-up, with a median time of first recurrence of 4.5 months (IQR: 2–16 months). *E. coli* was isolated in 5 children and *Klebsiella pneumoniae* in one child.

## Discussion

In Belgium, routine use of parenteral antimicrobial treatment in children with UTI has been recommended for decades because it was supposed to ensure optimal antimicrobial levels in these high-risk patients (13, 15). In the last two decades, at least four studies (9–12) showed that administration of oral and parenteral antibiotics in children with UTI had comparable efficacy in terms of duration of fever, disease recurrence and incidence of scars 6–12 months after infection. Accordingly, American (5), English (6), and Italian (7) national guidelines now provide similar recommendations to restrict hospitalization and parenteral antibiotic treatment to “complicated” UTI (e.g., febrile child appearing toxic, dehydrated, and/or unable to retain oral fluids and medication) and in cases where non-compliance is expected. The local patterns of susceptibility of coliforms to antimicrobial agents are clearly essential for the implementation of these guidelines in local operating procedures (8, 13). Substantial geographic variation in antibiotic resistance needs to be taken into account for the choice of the first-line empirical antimicrobial agents. Worldwide prevalence of antimicrobial resistance is increasing at a startling rate. A high rate of antibiotic resistance for *E. coli* strains, in particular, has been identified in Europe and North America (8, 16, 17). Urinary *E. coli* expressing *ESBLs* in Asia has already reached about 60% (18, 19), but remains fortunately low in Europe (13, 20).

In light of data in Belgium showing only a 2% resistance of *E. coli* strains to second and third generation cephalosporins (13), cefuroxime-axetil has been chosen as first line oral treatment in this study. Our results confirm the same low rate of resistance. However, 11% of children presented digestive symptoms and needed an antibiotic shift to the parenteral route. Surprisingly, these digestive side effects of cefuroxime-axetil in tablet form have only seldom been reported in adult literature (21). However, in pediatric populations the relatively bitter taste of cefuroxime-axetil suspension may reduce compliance by interfering with children's acceptance of oral therapy (22). Tolerance to cefuroxime-axetil in particular and to other antibiotics in general is however of crucial importance and must be taken into account as long as ambulatory oral treatment is concerned (22).

In this respect, it is interesting that in all four prospective and randomized trials which documented the efficacy of oral antibiotics in UTI children were treated as inpatients with treatment either administrated at the emergency department (9–11) or during hospitalization (12). Therefore, the efficacy of oral antibiotics as treatment for UTI in children clearly required validation in the context of outpatient therapy. Our single-center cohort suggests in a real life setting that ambulatory treatment of febrile UTI in children is possible when the prevalence of multi-resistant organisms is relatively low. Nevertheless, 21% of patients have been shifted from oral to intravenous treatment underlining the need for high compliance and close medical follow up for successful implementation of ambulatory treatment strategies.

Our pediatric hospital, located in the north of Brussels, acts as a first-line medical facility for socially and economically fragile patients, for whom adhesion to oral therapy and medical follow-up can be difficult to ascertain. The success of oral antibiotic treatment in this setting is clearly dependent on a public health system which reduces barriers to compliance and adequate follow-up. The situation in Belgium in this respect is relatively favorable (high level of general medical care with limited financial contribution by patients). Even so, 17% of children escaped medical controls during follow-up. The findings of the present study can therefore not be automatically transposed to other settings/countries.

The present study has several limitations. The overall cohort is of moderate size and the design of the study is not an intervention trial with a control group. In addition, children had no control urine culture after completion of antibiotic therapy for bacteriological confirmation of cure. Cefuroxime-axetil was used as the first-line antibiotic treatment. The palatability of the suspension, the dosing intervals (3 times per day) and duration of therapy (14 days) might have increased oral treatment failures. Oral third generation antibiotics are likely to be more effective but are unfortunately not commercially available in Belgium. Therefore, our results can only be interpreted for cefuroxime-axetil. An additional limitation of our study is the fact that conversion from oral to intravenous therapy is a subjective outcome which depends on clinical judgment of the treating pediatrician. We cannot exclude that some of the children with difficulties in taking oral antibiotics due to vomiting and/or persistent fever at the day 2 visit in spite of bacterial sensitivity might have been ultimately successfully treated by the oral route, mainly because these situations were considered at risk for treatment failure and potential renal damage. In a recent study Chaudhary et al. showed that substantial variation in admission rates exists for children with UTI in the United States (23). In addition, although hospitals with lower admission rates had higher revisit rates, those hospitals did not have an increase in revisits with subsequent admission, which differs from our results.

## Conclusion

This 2-year evaluation suggests that the implementation of international guidelines on oral treatment in febrile UTI is possible but requires caution. Home-treated children with cefuroxime-axetil need reevaluation during the scheduled treatment period since in our cohort more than one out of five of them were switched to parenteral therapy. In addition, a significant proportion of children can be lost to follow-up and local procedures are necessary to monitor and manage children who do not attend follow up visits during oral treatment.

## Author contributions

We certify that all authors are responsible for reported research. Each author listed on the manuscript has participated in the present work: EH conception, design of the work; statistical analysis, and interpretation of data. Drafting the work and revising it. HD conception, design of the work; the acquisition, statistical analysis, and interpretation of data. Drafting the work and revising it. BC, BA, KL, and SB interpretation of data, drafting the work and revising it. KW conception, design of the work; statistical analysis, and interpretation of data. Drafting the work and revising it. KI conception and design of study; data collection, statistical analysis and interpretation of data, drafting and revising of manuscript.

### Conflict of interest statement

The authors declare that the research was conducted in the absence of any commercial or financial relationships that could be construed as a potential conflict of interest. The reviewer FM and handling Editor declared their shared affiliation.

## Abbreviations

*E. coli*: Escherichia coli
ESBL: Extended-spectrum beta-lactamase
IQR: Interquartile range
UTI: urinary tract infection.

## References

[1] Sood A Penna FJ Eleswarapu S Pucheril D Weaver J Abd-El-Barr AE. Incidence, admission rates, and economic burden of pediatric emergency department visits for urinary tract infection: data from the nationwide emergency department sample, 2006 to 2011. J Pediatr Urol. (2015) 11:246.e1–8. 10.1016/j.jpurol.2014.10.005 26005017

[2] Doganis D Siafas K Mavrikou M Issaris G Martirosova A Perperidis G. Does early treatment of urinary tract infection prevent renal damage? Pediatrics (2007) 120:e922–8. 10.1542/peds.2006-2417 17875650

[3] Montini G Tullus K Hewitt I. Febrile urinary tract infections in children. N Engl J Med. (2011) 365:239–50. 10.1056/NEJMra1007755 21774712

[4] AFSSAPS Diagnostic et Antibiothérapie des Infections Urinaires Bactériennes Communautaires du nourrisson et de l'enfant. Recommandations de Bonne Pratique. Available online at: www.ansm.sante.fr

[5] Subcommittee on Urinary Tract Infection Steering Committee on Quality Improvement and Management Urinary Tract Infection Clinical Practice Guideline for the Diagnosis and Management of the Initial UTI in Febrile Infants and Children 2 to 24 Months. Pediatrics (2011) 128:595–610. 10.1542/peds.2011-1330 21873693

[6] Mori R Lakhanpaul M Verrier-Jones K. Diagnosis and management of urinary tract infection in children: summary of NICE guidance. BMJ (2007) 335:395–7. 10.1136/bmj.39286.700891.AD 17717369 PMC1952472

[7] Ammenti A Cataldi L Chimenz R Fanos V La Manna A Marra G Italian society of pediatric nephrology. febrile urinary tract infections in young children: recommendations for the diagnosis, treatment and follow-up. Acta Paediatr. (2012) 101:451–7. 10.1111/j.1651-2227.2011.02549.x 22122295

[8] Alberici I Bayazit AK Drozdz D Emre S Fischbach M Harambat J ESCAPE Study Group; PREDICT Trial. Pathogens causing urinary tract infection ns in infants: a European overview by the ESCAPE study group. Eur J Pediatr. (2015) 174:783–90. 10.1007/s00431-014-2459-3 25428232

[9] Hoberman A Wald ER Hickey RW Baskin M Charron M Majd M. Oral versus initial intravenous therapy for urinary tract infections in young febrile children. Pediatrics (1999) 104(1 Pt 1):79–86. 10.1542/peds.104.1.79 10390264

[10] Bocquet N Sergent Alaoui A Jais JP Gajdos V Guigonis V Lacour B. Randomized trial of oral versus sequential IV/oral antibiotic for acute pyelonephritis in children. Pediatrics (2012) 129:e269–75. 10.1542/peds.2011-0814 22291112

[11] Neuhaus TJ Berger C Buechner K Parvex P Bischoff G Goetschel P. Randomised trial of oral versus sequential intravenous/oral cephalosporins in children with pyelonephritis. Eur J Pediatr. (2008) 167:1037–47. 10.1007/s00431-007-0638-1 18074149

[12] Montini G Toffolo A Zucchetta P Dall'Amico R Gobber D Calderan A. Antibiotic treatment for pyelonephritis in children: multicentre randomised controlled non-inferiority trial. BMJ (2007) 335:386. 10.1136/bmj.39244.692442.55 17611232 PMC1955287

[13] Ismaili K Wissing KM Lolin K Le PQ Christophe C Lepage P. Characteristics of first urinary tract infection with fever in children: a prospective clinical and imaging study. Pediatr Infect Dis J. (2011) 30:371–4. 10.1097/INF.0b013e318204dcf3 21502928

[14] Duong HP Wissing KM Tram N Mascart G Lepage P Ismaili K. Accuracy of automated flow cytometry-based leukocyte counts to rule out urinary tract infection in febrile children: a prospective cross-sectional study. J Clin Microbiol. (2016) 54:2975–81. 10.1128/JCM.01382-16 27682127 PMC5121388

[15] Ferreiro C Piepsz A Nogarède C Tondeur M Hainaut M Levy J. Late renal sequelae in intravenously treated complicated urinary tract infection. Eur J Pediatr. (2013) 172:1243–8. 10.1007/s00431-013-2024-5 23677250

[16] Kahlmeter G. Prevalence and antimicrobial susceptibility of pathogens in uncomplicated cystitis in Europe: the ECO.SENS study. Int J Antimicrob Agents (2003) 22:49–52. 10.1016/S0924-8579(03)00229-2 14527771

[17] Zhanel GG Hisanaga TL Laing NM DeCorby MR Nichol KA Weshnoweski B. Antibiotic resistance in *Escherichia coli* outpatient urinary isolates: final results from the North American Urinary Tract Infection Collaborative Alliance (NAUTICA). Int J Antimicrob Agents (2006) 27:468–75. 10.1016/j.ijantimicag.2006.02.009 16713191

[18] Zowawi HM Harris PN Roberts MJ Tambyah PA Schembri MA Pezzani MD. The emerging threat of multidrug-resistant Gram-negative bacteria in urology. Nat Rev Urol. (2015) 12:570–84. 10.1038/nrurol.2015.199 26334085

[19] Duong HP Mong Hiep TT Hoang DT Janssen F Lepage P De Mol P. [Practical problems related to the management of febrile urinary tract infection in Vietnamese children]. Arch Pediatr. (2015) 22:848–52. 10.1016/j.arcped.2015.05.010 26143997

[20] Jacmel L Timsit S Ferroni A Auregan C Angoulvant F Chéron G. Extended-spectrum β-lactamase-producing bacteria caused less than 5% of urinary tract infections in a paediatric emergency centre. Acta Paediatr. (2017) 106:142–7. 10.1111/apa.13546 27542840

[21] Dellamonica P. Cefuroxime axetil. Int J Antimicrob Agents (1994) 4:23–36. 10.1016/0924-8579(94)90061-2 18611587

[22] Steele RW Thomas MP Bégué RE. Compliance issues related to the selection of antibiotic suspensions for children. Pediatr Infect Dis J. (2001) 20:1–5. 10.1097/00006454-200101000-00001 11176558

[23] Chaudhari PP Monuteaux MC Bachur RG. Management of urinary tract infections in young children: balancing admission with the risk of emergency department revisits. Acad Pediatr. (2018). 10.1016/j.acap.2018.05.011. [Epub ahead of print]. 29864523

